# Mutations in the 5’ NTR and the Non-Structural Protein 3A of the Coxsackievirus B3 Selectively Attenuate Myocarditogenicity

**DOI:** 10.1371/journal.pone.0131052

**Published:** 2015-06-22

**Authors:** Chandirasegaran Massilamany, Arunakumar Gangaplara, Rakesh H. Basavalingappa, Rajkumar A. Rajasekaran, Hiep Vu, Jean-Jack Riethoven, David Steffen, Asit K. Pattnaik, Jay Reddy

**Affiliations:** 1 School of Veterinary Medicine and Biomedical Sciences, University of Nebraska-Lincoln, Lincoln, Nebraska, United States of America; 2 Center for Biotechnology, University of Nebraska-Lincoln, Lincoln, Nebraska, United States of America; 3 Nebraska Center for Virology, University of Nebraska-Lincoln, Lincoln, Nebraska, United States of America; The Scripps Research Institute, UNITED STATES

## Abstract

The 5’ non-translated region (NTR) is an important molecular determinant that controls replication and virulence of coxsackievirus B (CVB)3. Previous studies have reported many nucleotide (nt) sequence differences in the Nancy strain of the virus, including changes in the 5’ NTR with varying degrees of disease severity. In our studies of CVB3-induced myocarditis, we sought to generate an infectious clone of the virus for routine *in vivo* experimentation. By determining the viral nt sequence, we identified three new nt substitutions in the clone that differed from the parental virus strain: C97U in the 5’ NTR; a silent mutation, A4327G, in non-structural protein 2C; and C5088U (resulting in P1449L amino acid change) in non-structural protein 3A of the virus leading us to evaluate the role of these changes in the virulence properties of the virus. We noted that the disease-inducing ability of the infectious clone-derived virus in three mouse strains was restricted to pancreatitis alone, and the incidence and severity of myocarditis were significantly reduced. We then reversed the mutations by creating three new clones, representing 1) U97C; 2) G4327A and U5088C; and 3) their combination together in the third clone. The viral titers obtained from all the clones were comparable, but the virions derived from the third clone induced myocarditis comparable to that induced by wild type virus; however, the pancreatitis-inducing ability remained unaltered, suggesting that the mutations described above selectively influence myocarditogenicity. Because the accumulation of mutations during passages is a continuous process in RNA viruses, it is possible that CVB3 viruses containing such altered nts may evolve naturally, thus favoring their survival in the environment.

## Introduction

Coxsackievirus B (CVB) is a positive-sense, single-stranded RNA virus belonging to the *picornaviridae* family [1,2]. CVB3, one of the six serotypes of the virus, commonly causes myocarditis and pancreatitis in affected individuals. The viral genome codes for a large polyprotein, which is proteolytically processed to generate the structural viral proteins (VP) 1, VP2, VP3, and VP4, and several nonstructural (NS) proteins [3]. The structural proteins are involved in the assembly of infectious virions, while the NS proteins are required for processing of the viral polyprotein and genome replication [3–5]. The viral genome lacks a 5’ cap structure that is required for translation and typically seen in most eukaryotic and many viral mRNAs [6,7]. Instead, the 5’ non-translated region (NTR) of the CVB3 genome contains an internal ribosome entry site, which mediates translation of viral mRNAs [8,9]. The 5’ NTR, accounting for 10% of the CVB3 genome [742 out of 7400 nucleotides (nts)], forms multiple secondary stem-loop structures and is known to harbor molecular determinants of viral pathogenicity [10–12]. For example, chimeric viruses resulting from replacement of the 5’ NTRs of CVB3 with corresponding regions from other viruses within the *Enterovirus* genus, such as echovirus and poliovirus, have been shown to exhibit reduced growth characteristics in permissive cell lines and/or lose pathogenicity [13,14].

Because CVB3 is an RNA virus and the viral RNA-dependent RNA polymerase lacks proof-reading activity [15], the viral genome is prone to errors. Several clinical isolates containing single and/or multiple nt changes have been reported with varying degrees of pathogenicity [16–21]. Experimentally, the Nancy strain of CVB3 is commonly used to study the immune mechanisms of viral myocarditis in various mouse models [22–25]. Our laboratory has been using CVB3 (Nancy strain procured from the American Type Culture Collection [ATCC]) to examine the molecular determinants of myocarditis induced by virus in myocarditis-susceptible A/J mice [23]. Because of the high error rate noted in the RNA viruses [26], we reasoned that the CVB3 genome might have undergone point mutation(s) during continuous passage under experimental conditions that could potentially alter viral pathogenicity *in vivo*. Therefore, we sought to derive an infectious clone of the viral genome such that a low passage virus containing minimal accumulation of mutations derived from the infectious clone would be more appropriate to study the immunopathogenesis *in vivo*. To this end, we constructed a full-length infectious clone of the CVB3 genome and determined the complete nt sequence. We identified three new nt substitutions that were not previously reported (C97U in the 5’ NTR; A4327G in the 2C [silent]; and C5088U, resulting in a P1449L substitution in the 3A). We observed that the infectious clone-derived virus induced significantly less severe myocardial damage but comparable pancreatitis in relation to the disease phenotype induced by the wild type (Wt) virus. Reversal of these mutations by generating three new infectious clones led us to identify a viral strain that regains myocarditogenicity, suggesting that the mutations described above might have collectively contributed to the attenuated myocarditis phenotype in the infected animals.

## Materials and Methods

### Ethics Statement

Six-to-eight-week-old A/J, C57BL/6 and BALB/c mice were procured from the Jackson Laboratory (Bar Harbor, ME). The mice were maintained according to the animal protocol guidelines of the University of Nebraska-Lincoln, Lincoln, NE; the Institutional Animal Care and Use Committee of the University approved the experimental protocol (Permit number: A3459-01; Protocol number: 973).

### Virus propagation, titration, and disease-induction

Vero cells (ATCC, Manassas, VA) grown to 80–90% confluence were infected with Wt CVB3 (Nancy strain, ATCC) as described previously [23]. Culture supernatants containing virus were harvested after the complete cytopathic effect (CPE) was confirmed, and the viral stocks were stored at -80°C until further use. After titrating the Wt and infectious clone-derived viruses, tissue culture infective dose (TCID)_50_ values were determined according to the Spearman-Karber method [27]. To compare the viral titers of Wt and infectious clone-derived viruses, Vero cells grown in 12-well plates were infected with viruses at multiplicity of infection (MOI), 0.1 and 3.0 in triplicates. After noting the CPE, viruses were harvested and titrated as above. Similarly, the viral titers were determined in the target organs namely, hearts and pancreata obtained from mice infected with Wt or infectious clone-derived virus. Briefly, small pieces of tissues (hearts or pancreata) from two mice each, were pooled and homogenized in sterile RPMI using the homogenizer, and the homogenate was centrifuged at 10,000 rpm for 15 minutes. After filtering the supernatant through a 0.2 μm sterile filter, the filtrate was used to infect monolayers of Vero cells grown in 6-well plates. The passage 1 (P_1_) viruses were harvested at 132 hours (heart samples), and 96 hours (pancreatic samples) postinfection. The P_1_ virus samples were then used to determine the differences in the viral titers between groups.

To infect mice, virus stock was diluted in 1x phosphate-buffered saline (PBS) to contain 2,000 TCID_50_/200 μl, and the inocula were administered intraperitoneally (i.p.), whereas the control (uninfected) mice received only 1x PBS. Animals were housed in filter-top cages (2 to 3 mice/cage) assembled with closed air-circulation. Cages containing the chow diet and waterers were changed once in 3 days until the termination of the experiment, and the animals had *ad libitum* access to food and water during the entire period of study. The animals were observed for clinical signs such as ruffled fur, isolation from the group/sluggish activity or self-mutilation and they were inspected twice a day, and body weights were taken daily. Additionally, alternative food- and fluid-source, trans gel diet (ClearH2O, Portland, ME) were placed in the cage floor as the animals started to show the clinical signs described above regardless of whether the companion animals within the cage were sick or not. Hearts and pancreata were collected from the animals that died naturally, and also from the animals euthanized on days 5, 7, 10 or at termination on day 21, postinfection. Animals were euthanized using CO_2_ chamber prefilled with 2% CO_2_, and spleens, lymph nodes, hearts and pancreata were then collected.

### Histopathology

Hearts and pancreata were fixed by immersion in 10% phosphate-buffered formalin, and processed for histological examination [28]. Briefly, three full-diameter heart sections were cut at 5 μm thickness and stained with hematoxylin and eosin (H and E). The sections were examined by the board (American College of Veterinary Pathologists)-certified pathologist, Dr. David Steffen blinded to treatment. Pathology scores were generated by enumerating the foci of inflammation, necrosis, mineralization, and fibrosis. The individual scores were used to compare the qualitative nature of the lesions. The total scores represent total foci of pathologic change across the three sections of heart. Multiple changes present in a single focus were counted as 1 in the total count [23,29]. The severity of pancreatic change was estimated as percent of tissue section involvement from one random section of pancreas. The nature of pancreatic lesions was noted as atrophy, inflammation, mineralization and necrosis or a combination of these [23,29].

### Derivation of full-length infectious cDNA clone of CVB3 and in vitro transcription

**Fig 1:**
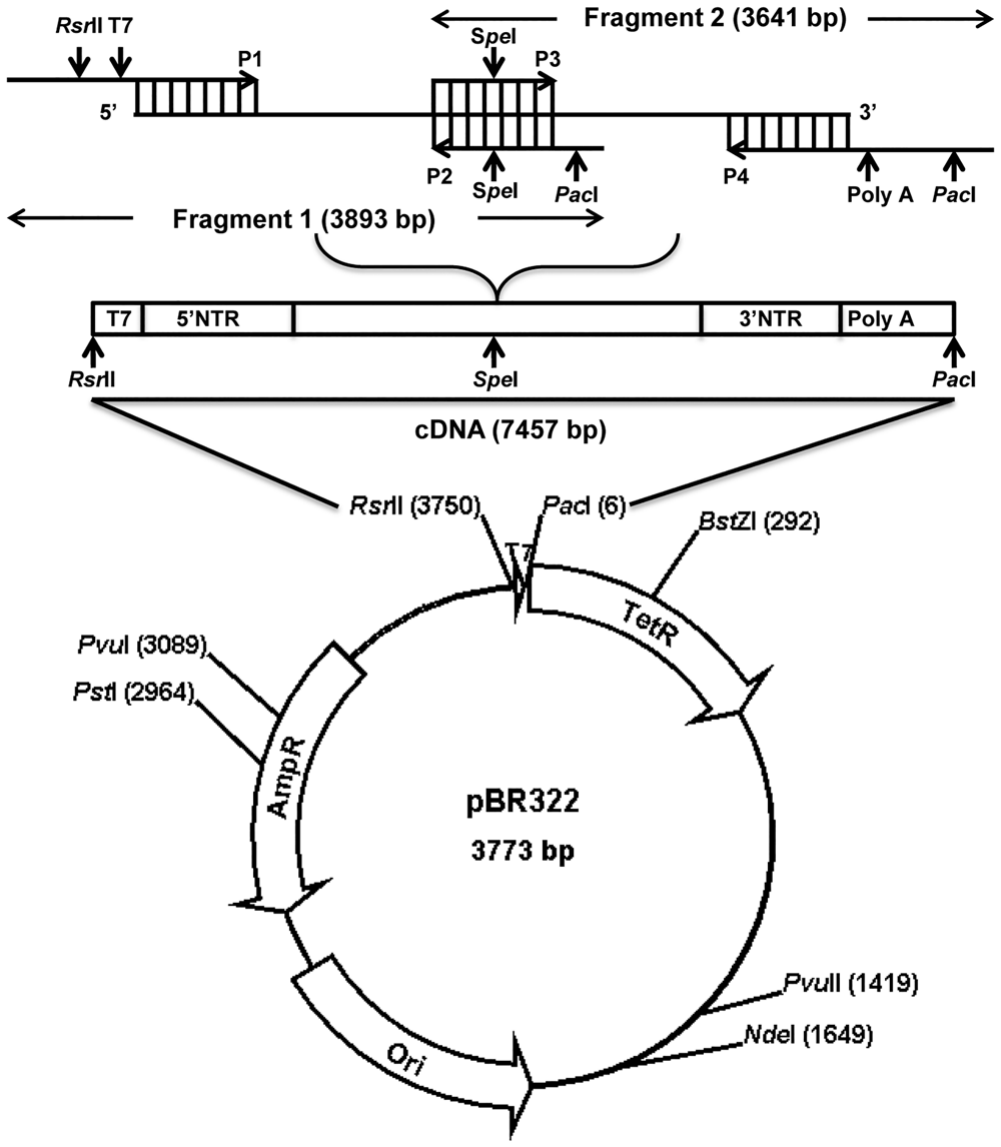
Derivation of infectious CVB3 cDNA clone. Using cDNA synthesized from the Wt CVB3 (Nancy) virus RNA, overlapping fragments 1 and 2 were amplified by PCR to generate a full-length cDNA clone. While R*sr*II and T7 RNA polymerase promoter sequences were inserted upstream of the 5’ NTR of fragment 1, the poly(A) tail (59 adenosine residues) and P*ac*I sequences were inserted downstream to the 3’ NTR of fragment 2. The fragments were cloned into the pBR322 vector and sequenced. Vector map was derived using SimVector software.

To generate the infectious clone, we first generated a stock virus (passage 1) from a well-isolated plaque of CVB3 (Nancy strain, ATCC) and then prepared RNA from the stock virus using Trizol LS reagent (Invitrogen, Carlsbad, CA). We synthesized full-length cDNA using Moloney murine leukemia virus-reverse transcriptase in a reaction mixture containing oligo-dT (Invitrogen) and performed PCR with this cDNA to amplify two fragments using high-fidelity *Pfu* polymerase (Agilent Technologies, Santa Clara, CA). The primer sets used in the preparation of these amplicons are described in Table 1; the amplicons were cloned into plasmid pBR322 in a stepwise manner (Fig 1). To facilitate *in vitro* RNA transcription, bacteriophage T7 RNA polymerase promoter was inserted in the 5’ end of P1 primer next to the R*sr*II site (Fig 1 and Table 1). The first fragment was digested with R*sr*II and P*ac*I and cloned into a modified pBR322 vector as described previously [30]. We digested this vector with S*pe*I and P*ac*I and gel-purified the plasmid DNA, into which we then cloned the second PCR fragment digested with S*pe*I and P*ac*I. This vector containing the full-length cDNA clone, hereafter designated, pBRCVB3 was sequenced leading us to identify three new nt changes that were not reported previously (Table 2). For *in vitro* transcription, the pBRCVB3 vector was linearized with P*ac*I, and the transcription reaction was performed using T7 RNA polymerase at 37°C as recommended by the manufacturer (Promega, Madison, WI).

**Table 1:** Primer sets used to generate infectious cDNA clone, pBRCVB3.

Primer	Sequence
Fragment 1	
	R*sr*II T7 promoter
P1	5’ATATATCGGACCG $\bar{\text{TAATACGACTCACTATAGGG}}$ **TTAAAACAGCCTG TGGGTTGAT**3’
	P*ac*I S*pe*I
P2	5’ATATTTAATTAA**TCTTGACCC****ACTAGT****GATTCTTTC**3’
Fragment 2	
	S*pe*I
P3	5’**GAAAGAATC****ACTAGT****GGGTCAAGA**3’
	P*ac*I
P4	5’TATATATTAATTAATTTTTTTTTTTTTTTTTTTTTTTTTTTTTTTTTTTTTTTTTTT TTTTTTTTTTTTTTTT**CGCACCGAATGCGGAGAATTTA**3’

Underlined nt, restriction enzyme sites; overlined nt, T7 promoter; bold nt, viral sequence.

**Table 2:** Nucleotide changes detected in the infectious clone, pBRCVB3.

Position	Region	nt changes*	Amino acids	References
35	5’ NTR	G addition		[21]
**97**	**5’ NTR**	**C to U**		**Not reported**
647	5’ NTR	C to U		[16]
667	5’ NTR	U to C		[16]
788	VP4	G to A	Gly to Arg	[16]
1180	VP2	A to G	Gly (silent)	[16,18,21]
1401	VP2	C to G	Thr to Ser	[16,18]
1402	VP2	G to C	Thr to Ser	[16,18]
1963	VP3	U to C	Asn (silent)	[16]
2201	VP3	G to A	Val to Ile	[16]
2438	VP3	G to C	Glu to Gln	[16]
2560	VP1	A to G	Glu (silent)	[16]
2593	VP1	C to U	Thr (silent)	[16]
2851	VP1	C to U	Asp (silent)	[16]
3346	VP1	A to G	Val (silent)	[16]
4177	2C	A to G	Glu (silent)	[16]
**4327**	**2C**	**A to G**	**Ala (silent)**	**Not reported**
**5088**	**3A**	**C to U**	**Pro to Leu**	**Not reported**
7026	3Dpol	U to C	Val to Ala	[16]
7334	3’ NTR	C to U		[16]

*Sequence comparisons were made between consensus sequence derived from the published sequences shown in parenthesis and pBRCVB3.

The first nt in column 3 represents consensus sequence, and the second nt represents pBRCVB3.

Bold text indicates nt changes not reported.

### Rapid amplification of cDNA ends (RACE)

To confirm viral sequences in the primers (P1 and P4) that flank the two ends of the full-length cDNA (Table 1), we performed RACE on plaque-purified Wt CVB3 RNA. For 5’ RACE, viral RNA was treated with tobacco acid pyrophosphatase to convert the 5’ tri-phosphate group to a mono-phosphate group, and the RNA 5’ adapter oligo was then ligated to Wt CVB3 RNA using RNA ligase (Epicenter, Madison, WI). cDNA was synthesized using the reverse primer internal to the viral sequence at the 5’ NTR (5’ AACCGCGTGAGCAGTCTATTG 3’). Using this cDNA mixture, we amplified a fragment of 266 bp by PCR using the 5’ adapter forward primer (supplied) and the reverse primer internal to the viral sequence as indicated above, then sequenced the PCR products. In 3’ RACE analysis, we first ligated the synthetic RNA 3’ adapter (5’ [phos]ACAAGCACACCCGAAGCGA CCAGCGGCAGA[phos] 3’) to the 3’ end of viral RNA using RNA ligase, modifying the oligo to include monophosphate groups at both the 5’ and 3’ ends to facilitate ligation and to avoid concatenation of oligos, respectively. After synthesizing cDNA using the reverse primer internal to the 3’ adapter (5’ CGCTGGTCGCTTCGGGTG 3’), we performed PCR with *Taq* DNA polymerase, using the forward primer 5’ TGCAACTCCCATCACCTGTACA 3’ (internal to the viral sequence) and the reverse 3’ adapter primer as indicated above. After purifying, the PCR products were cloned into pGEMT-Easy vector (Promega), and seventeen clones were then sequenced.

### Recovery of infectious virus from in vitro transcribed viral RNA

Vero cells grown to 80% confluence were trypsinized and washed twice with 1x PBS by centrifugation at 400xg for 6 minutes at room temperature (RT). Cells were resuspended in electroporation buffer (Biorad, Hercules, CA) to a cell density of 10x10^6^ cells/ml. The *in vitro* transcribed viral RNA (5μg) was taken in a 0.4 cm electroporation cuvette (Biorad), and cell suspension (2x10^6^ cells in 200 μl) was then added. The mixture was subjected to electroporation using Gene Pulser Xcell Electroporation System at 160V (Biorad), according to the manufacturer’s recommendations. The electroporated cells were aspirated gently and transferred to 6-well plates containing 2 ml of fresh EMEM/10% FBS prewarmed to 37°C. After 16 hours, the medium was removed; cells were washed with 1x PBS and replaced with 2 ml of fresh EMEM/2% FBS. Cells were incubated up to 4 days, and as the CPE became evident, supernatants containing virus were harvested, passaged and titrated, and stored as above.

### Creation of new infectious clone-derived viruses to revert the mutations noted in pBRCVB3 virus

After sequencing and ascertaining the presence of three new nt changes—C97U in the 5’ NTR, and A4327G and C5088U in the NS proteins 2C and 3A, respectively in pBRCVB3 virus (Table 2)–we sought to revert these mutations by generating three new infectious cDNA clones using pBRCVB3 as a backbone (S1 Fig). These include: 1) U97C fixed (pBRCVB3/97); 2) G4327A and U5088C fixed (pBRCVB3/4327_5088); and 3) U97C, G4327A and U5088C fixed (pBRCVB3/97_4327_5088). Derivation of these clones required the synthesis of gene segments to be incorporated into pBRCVB3 (Genscript, Piscataway, NJ). To generate pBRCVB3/97, a 290 bp fragment containing the nt C at the desired position (97th) was cloned into R*sr*II (5’) and B*st*BI (3’) sites in pBRCVB3. Likewise, a fragment (2036 bp) bearing the desired nt change (A/4327 and C/5088) was cloned into pBRCVB3 at B*ss*HII (5’) and B*st*EII (3’) sites to obtain pBRCVB3/4327_5088. Finally, pBRCVB3/97_4327_5088 was obtained by cloning the fragment (2036 bp) released from pBRCVB3/4327_5088 into pBRCVB3/97 after digesting with B*ss*HII and B*st*EII. The *in vitro* transcription procedures and the recovery of infectious viruses were as described above.

### Immunofluorescence

To verify infectivity of viruses derived from the infectious clones, we used CVB3 anti-serum (ATCC) that allowed us to detect cells expressing viral proteins based on immunofluorescence [21]. Briefly, Vero cells (2x10^5^ cells/ml/well) were grown in triplicate on sterile coverslips in 12-well plates overnight at 37°C. The monolayers were washed with sterile 1x PBS and infected with the Wt (positive control), pBRCVB3, pBRCVB3/97, pBRCVB3/4327_5088, and pBRCVB3/97_4327_5088 at MOI of 0.5 for 1.5 hours. After adsorption, inoculum was aspirated and replaced with EMEM/2% FBS. Cells grown in medium alone were used as negative controls. After incubating for 12 hours at 37°C, cells were washed and fixed in methanol/acetone (1:1) at RT for 40 minutes. Cells were then washed thrice with sterile 1x PBS and incubated with anti-CVB3 serum (1:200) in PBS containing 2.5% bovine serum albumin at RT for one hour. After five washings with PBS/Tween 20 (PBST, 0.05%), cells were incubated with fluorescein isothiocyanate (FITC)-conjugated secondary rabbit, anti-horse IgG (1:1000; Sigma-Aldrich, St. Louis, MO) at RT for one hour. Finally, coverslips containing the cells were washed, mounted and visualized under Nikon A1-Eclipse 90i confocal microscope system (Nikon Instruments Inc-Americas, Melville, NY). Images were acquired sequentially at an excitation/emission wavelength of 561.5 nm/553–618 nm laser-setup for pseudocolored “green” channel (60x).

### Statistical analysis

Viral titers were determined on days 4 and 6 postinfection for Wt CVB3 and its variants. The data were tested not significant for normality by Lilliefors test [31]. Based on a non-normal distribution, the Kruskal-Wallis test [32] was applied, with subsequent contrasts between variants with multiple test correction via Tukey’s honestly significant difference criterion [33]. A Generalized Linear Mixed Model was created to determine differences in the body weights between groups. Several covariance structures were used to calculate the relative quality for the model as determined by the Akaike information criterion [34] corrected for finite sample sizes (605.53 ≤ AICc ≤ 673.18). The Ante-Dependence had the best fit (AICc = 605.53), and it was selected. The survival curves were analyzed via Cox Proportional Hazards Regression [35]. Differences in the medians of myocardial lesions were analyzed by the non-parametric Wilcoxon rank sum test [36]. One-way MANOVA analysis was performed to evaluate differences in pancreatitis. All statistical tests were performed in MATLAB (R2014b, The Mathworks Inc., Natick, MA, USA), except the test on body weights, which was performed using GLIMMIX procedure in SAS (version 9.3, SAS Institute Inc., Cary, NC, USA).

## Results and Discussion

CVB3 (Nancy) is a commonly used laboratory strain to study the immunopathogenesis of viral myocarditis in various mouse models [22–25]. To verify the pathogenicity of the Wt virus, we used A/J mice, which are highly susceptible to CVB3 infection [23,37,38]. We first noted that the mice infected with 50 TCID_50_/animal developed myocarditis and pancreatitis accompanied by necrosis as reported previously [22,23,25].

Since CVB3 is an RNA virus and the viral genome is prone to errors during continuous passage under experimental conditions [39], we sought to generate the infectious cDNA clone of the virus to obtain a virus with a conserved genome sequence for further experimentation. To accomplish this, we assembled the full-length cDNA clone, pBRCVB3, from two RT-PCR-derived amplicons spanning the entire length of the viral genome. The PCR fragments were cloned in the pBR322 vector to generate the full-length clone (Fig 1). Sequencing of three individual full-length clones resulted in the derivation of the consensus viral sequence bearing 7399 nts (NCBI AC_JX312064.1).

Since we had designed primers (P1 to P4; Table 1) to construct an infectious cDNA clone based on sequence information first deposited in the NCBI database [18], it was necessary to verify the authentic sequences of the viral genome at the termini, as well as the viral sequences spanning the primers P2 and P3 (Fig 1). By employing the RACE technique, we obtained sequences of the termini of the viral genome. Using RNA isolated from a virus stock prepared from a single plaque-purified Wt virus, we determined that the viral sequences at both the 5’ and 3’ termini were identical to those present in the P1 and P4 primers (Table 1). Although, based on the published literature [16], we had incorporated 59 adenosine residues at the end of the 3’ NTR in the infectious clone, our 3’ RACE analyses of 17 different clones revealed variable poly(A) tail lengths ranging in size from 19 to 52 adenosine residues. Such variations in the poly(A) tail length have been reported previously [16]. The 3’ RACE analysis also revealed the presence of one additional G residue at the extreme 3’ terminus prior to the poly(A) tail, thus making the full-length parental viral genome to be 7400 bases. Because it had previously been shown that the presence or absence of this additional G residue in the viral genome does not influence infectivity or pathogenicity of the virus [16,18,40,41], we did not incorporate this residue into our infectious cDNA clone (pBRCVB3). Sequence analyses of the regions spanning the P2 and P3 primers also revealed authentic sequences of the viral genome.

We then compared the sequence of our infectious clone, pBRCVB3, with previously published CVB3 (Nancy strain) genome sequences [16,18,21]. We noted changes in 15, 61, and 23 nts corresponding to AC_M33854.1, AC_M16572.1, and AC_JN048468.1, respectively (S1 Table), but such changes are to be expected [17,18,21]. We then derived a consensus sequence from the above three and compared it to the pBRCVB3 sequence, leading us to identify 20 nts that were different. These included five nts in the NTRs (four in the 5’ NTR and one in the 3’ NTR); 11 nts in the structural protein regions (one in VP4, three each in VP2 and VP3, and four in VP1); and four nts in the NS protein regions (two in 2C and one each in 3A and 3D; Table 2). Three of these have not been previously reported: C97U in the 5’ NTR; A4327G, silent, in the 2C protein; and C5088U, resulting in P1449L substitution, in the 3A protein (Table 2).

Nucleotide variations in the structural and NS protein regions, including the 3’ NTR, may or may not alter the growth characteristics and pathogenicity of the virus [20,21,41–44]. However, alterations in the 5’ NTR have been identified as important molecular determinants of viral virulence [10–12]. For example, a single nucleotide substitution of U to C, at position 234 attenuates cardiovirulence in mice [44]. Conversely, in a separate study, when 13 naturally occurring isolates of CVB3 were tested for their pathogenicity, only two isolates were found to be pathogenic—but all of them had U at position 234, suggesting that nt variations in other positions in the 5’ NTR, if any, may influence virulence in the natural isolates [45]. Likewise, A to U nt changes at positions 580 or 690 within the 5’ NTR were found not to be critical for viral attenuation [46]. Furthermore, comparison of sequences of the 5’ NTRs between myocarditic and non-myocarditic CVB3 strains by computational analysis revealed variations in 23 positions; of these, 14 changes located within the four stem-loops of the secondary structures of viral RNA were predicted to influence cardiovirulence [47]. In our infectious clone, one of the four nt changes noted in the 5’ NTR at position 97 (C to U) had not been previously reported, leading us to evaluate the virulence and pathogenic properties of the infectious cDNA clone-derived virus (pBRCVB3 virus).

**Fig 2:**
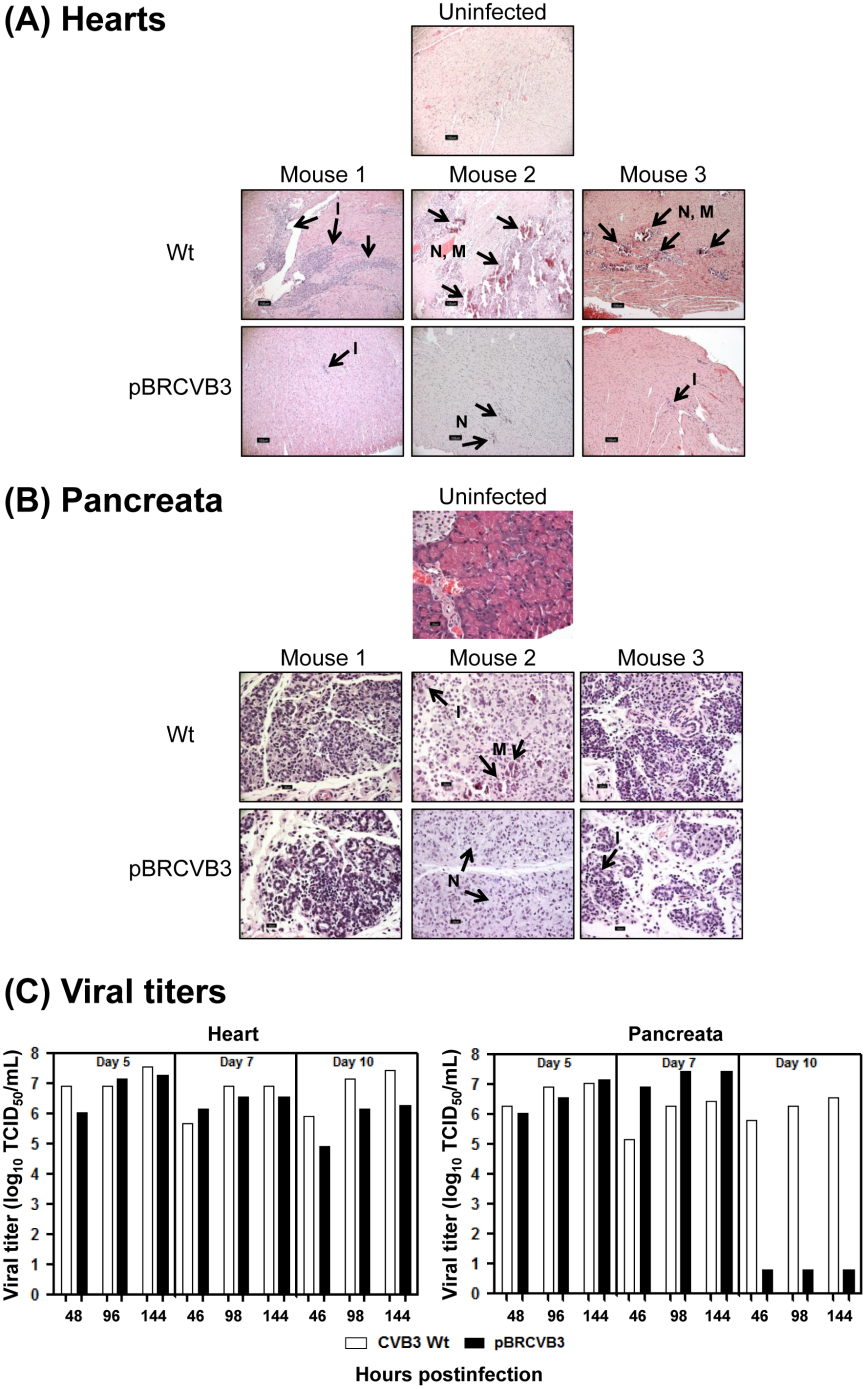
Myocarditis severity is attenuated in mice infected with the infectious clone-derived virus, pBRCVB3. **Histology.** A/J mice were infected with Wt virus or the virus derived from pBRCVB3 i.p., and hearts and pancreata were harvested at termination on day 21 postinfection or as the animals died or euthanized. The tissues were examined by H and E staining. Representative sections from three individual animals infected with Wt virus or pBRCVB3 virus are shown (n = 15 to 17 mice per group), whereas the sections obtained from uninfected animals were used as controls. (**A) Hearts.** Top panel (control group): heart section from uninfected animal without inflammation, mineralization or necrosis; middle panels (Wt virus-infected): extensive infiltrates of macrophages and lymphocytes into the myocardium filling areas of myocardial cell loss/necrosis (Mouse 1), extensive large foci of necrosis and mineralization surrounded by inflammation consisting of macrophages and lymphocytes (Mouse 2), multiple small foci of necrosis and mineralization infiltrated by a few inflammatory cells (Mouse 3); bottom panels (pBRCVB3 virus-infected): small focal inflammatory infiltrates into myocardium (Mouse 1), a small foci of necrosis in the myocardium (Mouse 2), small foci of lymphocytic inflammation (Mouse 3). (**B) Pancreata.** Top panel (control group): pancreas from an uninfected animal showing intact islets; middle panels (Wt virus-infected): diffuse atrophy with scattered lymphocyte infiltrates (Mouse 1), acute necrosis, mineralization and inflammation (Mouse 2), diffuse marked atrophy with mild diffuse inflammation (Mouse 3); bottom panels (pBRCVB3 virus-infected): diffuse atrophy and lymphocytic infiltrates (Mouse 1), diffuse necrosis with pyknotic nuclei (Mouse 2), diffuse atrophy with mild inflammation (Mouse 3). Arrows indicate the lesions (N, necrosis; M, mineralization; and I, inflammation). Original magnification: 10x (hearts) and 40x (pancreata). (**C) Viral titers.** Hearts and pancreata obtained from the above groups on days 5, 7 and 10 postinfection were processed for determining the viral titers as described in the ‘methods’ section and the titers were compared between groups. Left panel, hearts; right panel, pancreata.

To determine the virulence of pBRCVB3 virus, we first recovered the virus from the infectious clone following transfection of Vero cells with *in vitro*-transcribed RNA. After passaging the virus twice in cultures (similar to Wt virus), we sought to determine its pathogenicity *in vivo*. Groups of A/J mice were infected with 2000 TCID_50_/animal, and on days 5, 7, 10 or 21 postinfection, animals were euthanized; hearts and pancreata were evaluated for inflammatory changes, and the lesions were compared with uninfected animals. Unexpectedly, in animals infected with the infectious clone-derived virus, myocarditis was significantly attenuated (Table 3). As shown in Fig 2A, myocarditis in mice infected with the Wt virus showed widespread lesions containing necrosis, mineralization, and lymphocytic infiltrates as opposed to occasional lesions in pBRCVB3 virus-infected animals. Pancreatitis, however was consistently present in both groups of animals with comparable severity as indicated by atrophic changes, necrosis and inflammatory infiltrates (Fig 2B; Table 4). The finding that pBRCVB3 virus-induced pancreatitis is comparable to Wt virus suggests that the virus did reach the target tissues in infected animals. But, why then attenuated myocarditogenicity? One possible reason could be that the tropism of pBRCVB3 virus containing the nt changes (C97U, A4327G, and C5088U) might have been altered for the heart. To address this possibility, we determined the viral titers from hearts and pancreata obtained from animals infected with Wt virus or pBRCVB3 virus on days 5, 7 and 10 postinfection. The virus was found to be present in hearts from animals infected with pBRCVB3 virus similar to Wt virus-infected group as the viral titers were comparable at all the time-points postinfection (Fig 2C, left panel). Likewise, viral titers from pancreata from both the groups were also comparable except that pBRCVB3 virus could not be recovered on day 10 postinfection (Fig 2C, right panel).

**Table 3:** Histological evaluation of hearts in mice infected with different CVB3-variants.

Group	Incidence	Mortality^†^	Myocardial lesions
CVB3 Wt	14 / 15 (93.33)	8 / 12 (66.67)	68.47 ± 11.18
pBRCVB3	13 / 17 (74.47)	5 / 11 (45.45)	3.71 ± 1.21^a^
pBRCVB3/97	9 / 20 (45.00)	7 / 20 (35.00)	8.50 ± 3.88^a^
pBRCVB3/4327_5088	7 / 13 (53.85)	7 / 13 (53.85)	26.38 ± 11.75^a^
pBRCVB3/97_4327_5088	11 / 12 (91.67)	4 / 12 (33.33)	47.17 ± 18.95^b^

^†^represents natural deaths;

() indicates percentages;

^a,^ denotes significant differences in comparison with the CVB3 Wt (p<0.03);

^b,^ denote significant differences in comparison with the pBRCVB3/97 (p = 0.04)

**Table 4:** Histological evaluation of pancreata in mice infected with different CVB3-variants.

Group		Lesions			
	Incidence	Atrophy	Inflammation	Necrosis	Mineralization
CVB3 Wt	17 / 18 (94.44)	14 / 18 (77.78)	17 / 18 (94.44)	6 / 18 (33.33)	6 / 18 (33.33)
pBRCVB3	17 / 17 (100.0)	13 / 17 (76.47)	15 / 17 (88.24)	8 / 17 (47.06)	7 / 17 (41.18)
pBRCVB3/97	20 / 20 (100.0)	16 / 20 (80.00)	15 / 20 (75.00)	5 / 20 (25.00)	5 / 20 (25.00)
pBRCVB3/4327_5088	11 / 11 (100.0)	7 / 11 (63.63)	11 / 11 (100.0)	5 / 11 (45.45)	4 / 11 (30.76)
pBRCVB3/97_4327_5088	9 / 9 (100.0)	9 / 9 (100.0)	6 / 9 (66.66)	3 / 9 (33.33)	3 / 9 (33.33)

() represents percentages

We next asked whether the myocarditis-attenuating phenotype of pBRCVB3 virus can be recapitulated in other mouse strains. To address this question, groups of C57BL/6 and BALB/c mice were infected with Wt virus or pBRCVB3 virus, and hearts and pancreata were examined for histological changes. The data revealed that myocarditis was completely absent in animals infected with pBRCVB3 virus, whereas pancreatitis-severity was comparable in both C57BL/6 and BALB/c mice (Figures A and B of S2 Fig; S2 and S3 Tables). Furthermore, by evaluating the viral titers from the target organs, we noted that the virus could be recovered from hearts of both C57BL/6 and BALB/c mice on different days (5, 7 and 10) postinfection with the Wt virus, whereas the pBRCVB3 virus could only be recovered up to day 5 and day 7 from C57BL/6 and BALB/c mice, respectively (Figure C of S2 Fig). Of note, while the Wt virus could be isolated up to day 5 from the pancreata derived from both C57BL/6 and BALB/c mice, the pBRCV3 virus could be isolated up to day 7 from both the strains (S2C Fig). Taken together, the data suggest that the pBRCVB3 virus carrying the three nt substitutions (C97U, A4327G and C5088U) represent an attenuated variant of CVB3 with a selective loss of myocarditis-inducing ability.

**Fig 3:**
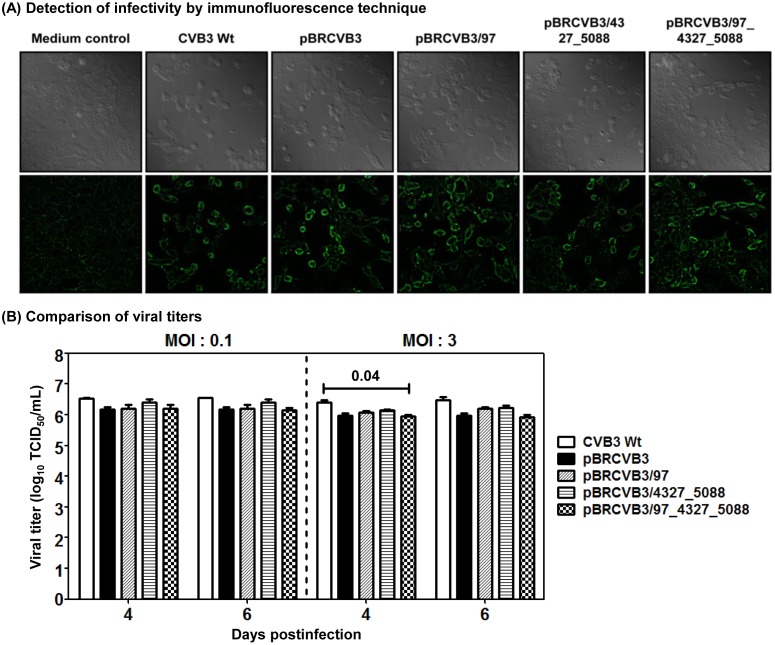
Infectivity of viruses derived from various infectious cDNA clones. **(A) Detection of infectivity by immunofluorescence technique.** Vero cells grown in monolayers on coverslips were cultured further in the medium alone or infected with EMEM containing CVB3 Wt, pBRCVB3, pBRCVB3/97, pBRCVB3/4327_5088, or pBRCVB3/97_4327_5088 in 12-well plates. After 12 hours of incubation at 37°C, cells were fixed, incubated with anti-CVB3 serum followed by secondary FITC-conjugated IgG to detect the viral antigens. The coverslips were washed and mounted and examined by Laser Scanning Confocal Microscope. Top panel: phase-contrast images. Bottom panel: immunofluorescence images. Original magnification: 60x. **(B) Comparison of viral titers.** Monolayers of Vero cells were grown to 80% confluence in 12-well plates. Triplicate wells were infected with MOI of 0.1 or 3 of P_2_ viruses of CVB3 Wt, pBRCVB3, pBRCVB3/97, pBRCVB3/4327_5088, or pBRCVB3/97_4327_5088. After noting the CPE, the respective viral supernatants were harvested and the viral titers (TCID_50_) were determined by Spearman-Karber method. Mean ± SEM values are shown (n = 3).

To test this hypothesis, we derived three new infectious cDNA clones using pBRCVB3 as a backbone by reverting the mutations (S1 Fig). These include pBRCVB3/97 (U97C), pBRCVB3/4327_5088 (G4327A and U5088C), and pBRCVB3/97_4327_5088 (U97C, G4327A and U5088C). First, we recovered the infectious viruses from all the clones, and after passaging twice in Vero cells, ascertained the expression of viral proteins by immunofluorescence using Wt virus as a positive control, and media containing uninfected cells as negative controls. Fig 3A shows that Vero cells infected with the Wt virus and the clone-derived viruses exhibited viral protein expression, but not in medium control, suggesting that all viral variants retained the infectivity similar to the Wt virus. We next determined the viral titers at MOIs of 0.1 and 3, and the data revealed no significant differences between groups except that the titers obtained from pBRCVB3/97_4327_5088 virus tended to be lower than those obtained from the Wt virus on day 4, only at MOI of 3 (Fig 3B). Together, the data suggest that the nt variations present in the three new infectious clones do not adversely affect viral recovery and their corresponding viruses retain viral infectivity.

**Fig 4:**
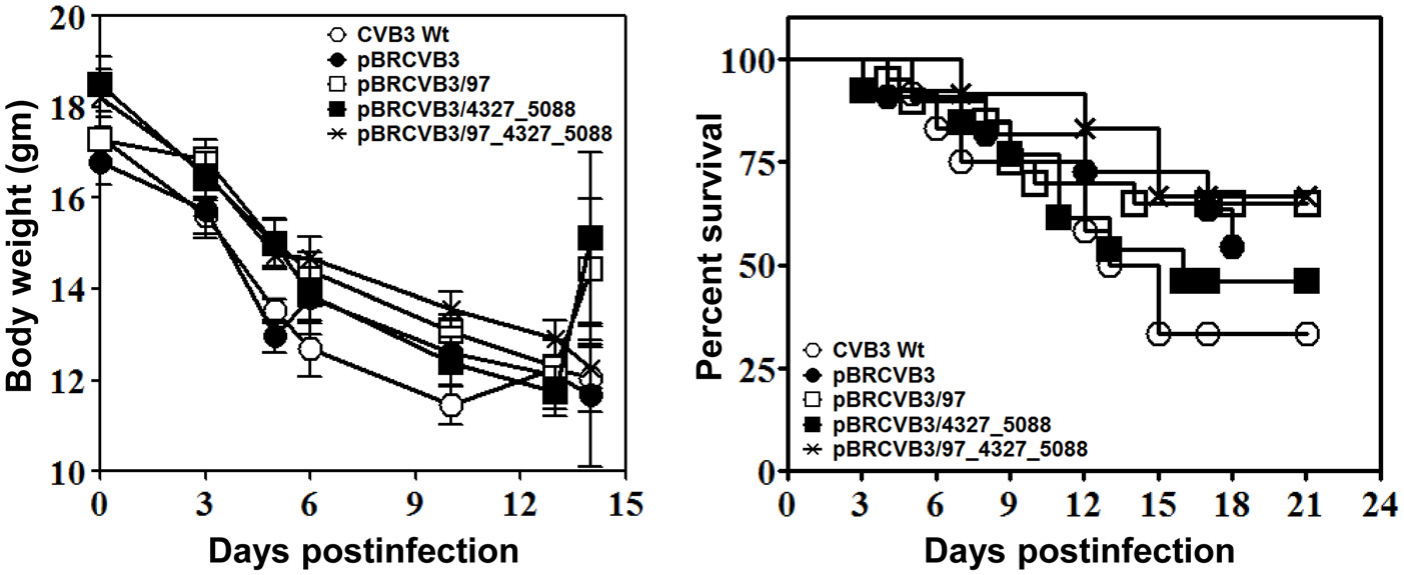
Body weight loss and mortalities in mice infected with viruses derived from different infectious cDNA clones. A/J mice infected with the indicated viruses were monitored for loss of body weight and mortalities. Body weights measured in grams on days 0, 3, 5, 6, 10, 13 and 14 were plotted. Mean ± SEM values derived from two individual experiments, each involving 6 to 7 animals per group, are shown (left panel). The number of animals that died in each group was noted (n = 11–20 mice per group), and survival curves were then derived using GraphPad Prism 6 software (right panel).

To determine virulence of the newly generated viruses, groups of animals were infected with the Wt virus or its variants and monitored for loss of body weight and mortalities. Generally, acutely infected animals lose body weight and die within approximately 10 days postinfection, and not surprisingly, variations in loss of body weight were noted between groups over a period of time (p = 0.04). As shown in Fig 4 (left panel), the body weight loss tended to be more dramatic in animals infected with the Wt virus compared with other groups, in particular in animals infected with the viruses derived from pBRCVB3/97 (p = 0.04) and pBRCVB3/97_4327_5088 (p = 0.05). Likewise, mortalities as shown by survival curves also tended to be greater in animals infected with the Wt virus (67%) as compared with other groups (33 to 54%), but the differences were insignificant (Fig 4, right panel; Table 3). At termination on day 21 (or as animals died), hearts and pancreata from the infected animals were examined to verify the severity of myocarditis (Table 3) and pancreatitis (Table 4).

**Fig 5:**
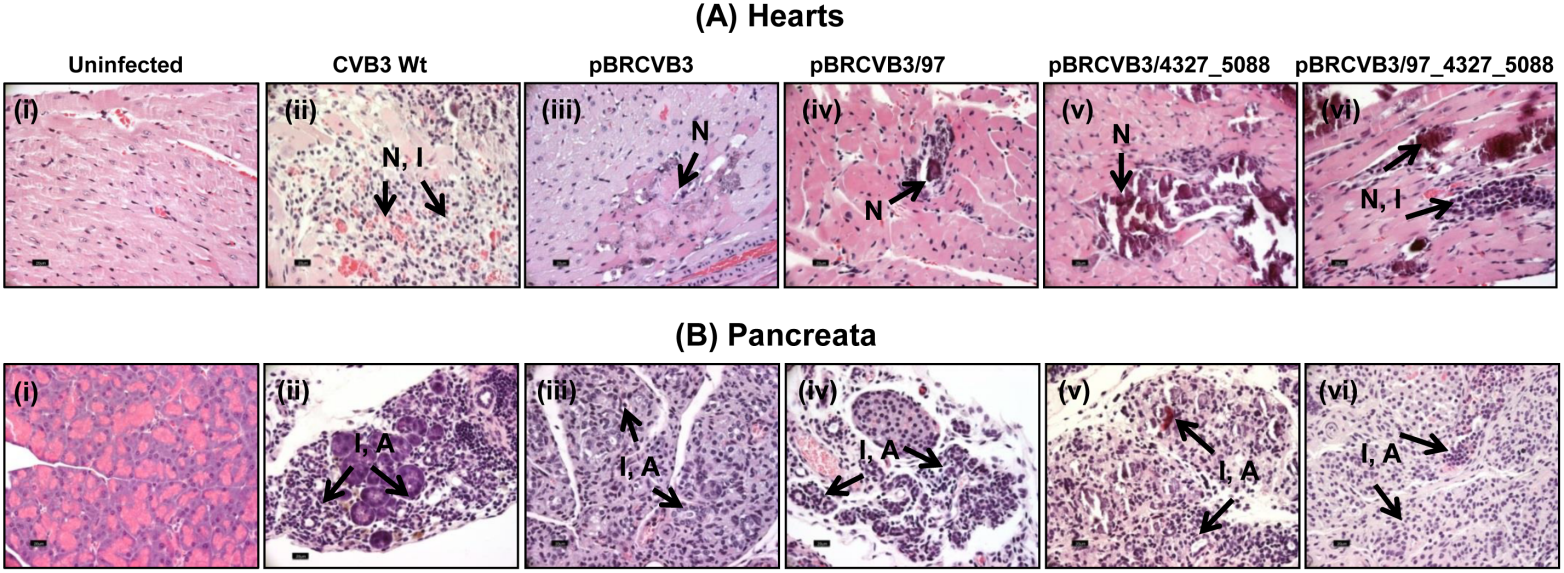
Reversion of mutations in parental infectious clone-derived virus, pBRCVB3, rescues the myocarditis phenotype. Groups of 6- to 8-week old female A/J mice were infected with Wt virus or pBRCVB3, pBRCVB3/97, pBRCVB3/4327_5088, or pBRCVB3/97_4327_5088 virus. At termination on day 21 postinfection or as the animals died, hearts and pancreata were collected for histological evaluation of inflammatory changes by H and E staining. **(A)** Hearts from uninfected mice showed no apparent changes (i). Wt virus-infected mice showed widespread hemorrhagic necrosis and lymphocytic infiltrations (ii), whereas pBRCVB3 virus-infected mice had focal myocardial necrosis (iii), as opposed to hemorrhagic necrosis seen in mice infected with pBRCVB3/97 and pBRCVB3/4327_5088 viruses (iv and v) and hemorrhagic necrosis and lymphocytic infiltrations in pBRCVB3/97_4327_5088 virus-infected mice (vi). **(B)** Pancreata from the corresponding groups as described above are shown (uninfected normal pancreas [i] and widespread atrophic changes, necrosis and lymphocytic infiltrations [ii to vi]). Arrows indicate the lesions (N, necrosis; I, inflammation; and A, atrophy). Original magnification: 40x. n = 11 to 20 mice per group.

All animals infected with the Wt virus or its variants showed myocardial damage with an incidence of 45% to 93%. The histological changes include inflammation, necrosis, mineralization and fibrosis, with necrosis being a prominent lesion. Similarly, pancreatitis was consistently seen in all the groups with an incidence of 94% to 100%. While the hearts and pancreata from uninfected mice showed normal structural features (Fig 5Ai, and 5Bi), animals infected with the Wt virus showed lymphocytic infiltrates and hemorrhagic necrosis in the myocardium including widespread atrophic changes in the pancreatic acini and pancreatic necrosis, all of which are expected changes in CVB3 infection (Fig 5Aii, and 5Bii) [22,23]. Likewise, the animals infected with pBRCVB3 virus also had severe pancreatitis similar to Wt virus-infected animals (Fig 5Biii), but the myocarditis severity was significantly low (Fig 5Aiii; Tables 3 and 4). In contrast, animals infected with the viruses generated from the newly derived infectious clones, pBRCVB3/97, pBRCVB3/4327_5088, or pBRCVB3/97_4327_5088, showed pancreatitis similar to that of Wt virus (Fig 5Biv, 5Bv and 5Bvi), but the severity of myocarditis varied in decreasing order from pBRCVB3/97_4327_5088 (Fig 5Avi), followed by pBRCVB3/4327_5088 (Fig 5Av), then pBRCVB3/97 viruses (Fig 5Aiv), leading us to make three observations: 1) myocarditis severity was greater in animals infected with the Wt virus, compared to those infected with the viruses derived from the parental infectious clone (pBRCVB3; p = 0.0013) or two of its newly derived variants, pBRCVB3/97 (p = 0.0001) or pBRCVB3/4327_5088 (p = 0.0248), but not those in the pBRCVB3/97_4327_5088 virus-infected group (Table 3); 2) the severity of myocarditis induced with pBRCVB3/97 or pBRCVB3/4327_5088 viruses did not differ from that induced with pBRCVB3 virus, suggesting that reversion of the mutations U97C and G4327A and U5088C in the respective clones individually did not influence the disease outcome; and 3) the disease severity induced by pBRCVB3/97_4327_5088 virus differed from that induced by pBRCVB3 virus (Table 3), indicating that reversion of two mutations (U97C and U5088C) together was critical to regain myocarditogenicity. However, A4327G in the parental clone (pBRCVB3) did not seem to contribute to the disease pathogenesis since it is a silent mutation, but the mechanistic basis for such an alteration in disease severity remains to be determined.

In summary, we have described construction of an infectious clone of CVB3, whose genomic sequence analysis revealed three novel nt changes, one of which (C97U) was found in the 5’ NTR, and the other two, A4327G, and C5088U, were noted in 2C and 3A proteins, respectively, of CVB3. By characterizing the pathogenic attributes of virus generated from the infectious clone, we noted that two mutations—C97U in the 5’NTR, and C5088U in the NS 3A protein—might have contributed to the attenuated myocarditis phenotype associated with pBRCVB3 virus. Although the other mutation, A4327G in 2C protein, is a silent mutation, we decided to revert it anyway because the CVB3 containing this mutation had not been previously reported. Literature indicates that both attenuated and cardiovirulent strains of CVB3 can replicate and generate infectious viral particles *in vitro*, but they may or may not be pathogenic *in vivo* [12,13,44]. The finding that the pBRCVB3 virus bearing C97U and C5088U showed selective attenuation of disease in the heart but not in pancreas suggests that the occurrence of mutations can differentially contribute to viral virulence by organ type. The availability of such tools may permit us to determine the molecular mechanisms of differential organ-specific disease phenotypes in future studies. It is possible that CVB3 strains possessing such altered nts may evolve naturally, and such alterations may favor the survival of avirulent strains of viruses in the environment.

## Supporting Information

S1 Fig Location of mutations in the infectious cDNA clones. Left panel indicates the location of nts U, G and U at positions, 97, 4327 and 5088, respectively, in parental infectious cDNA clone. Right panel indicates the location of reverted nts U to C, G to A, and U to C, at positions, 97, 4327 and 5088, respectively, in the newly generated infectious cDNA clones. Vector maps were derived using SnapGene software.
(TIF)

S2 Fig The attenuated myocarditis-phenotype induced with the infectious clone-derived virus, pBRCVB3 was recapitulated both in C57BL/6, and BALB/c mice. Groups of C57BL/6 and BALB/c mice were infected with Wt virus or pBRCVB3 virus. Animals were euthanized on days 5, 7, and 10 postinfection, and hearts and pancreata were collected for histological examination by H and E staining. **(Figure A) Hearts.** Section from C57BL/6 mice infected with Wt virus showing a necrotic fiber (arrow) surrounded by a few lymphocytes (i), as opposed to apparently normal heart from pBRCVB3 virus-infected animal (ii). Similarly, BALB/c mice showed hyper-eosinophilic necrotic fibers with pyknotic nuclei surrounded by a few lymphocytes (arrow; iii), whereas animals infected with pBRCVB3 virus had apparently normal hearts (iv). **(Figure B) Pancreata.** Four representative sections are shown, two each for C57BL/6 and BALB/c mice. C57BL/6: Wt virus-infected animal showing diffuse necrosis and inflammation (i); and pBRCVB3 virus-infected animal showing diffuse necrosis, inflammation, and mineralization (ii). BALB/c mice: Animal infected with Wt virus showing foci of lymphocytic inflammation in atrophied pancreas (iii), as opposed to diffuse inflammation with atrophied pancreas in pBRCVB3 virus-infected mouse (iv). Original magnification, 40x. n = 6 per group. **(Figure C) Viral titers.** Hearts and pancreata harvested from the above groups were processed for determining the viral titers as described in the methods section. Top panels: Heart and pancreata from C57BL/6 mice; bottom panels: Heart and pancreata from BALB/c mice.
(TIF)

S1 Table List of nucleotide changes detected in pBRCVB3 in comparison with other Nancy strains of CVB3. (DOCX)

S2 Table Histological evaluation of hearts from C57BL6 and BALB/c mice infected with CVB3 Wt and pBRCVB3. (DOCX)

S3 Table Histological evaluation of pancreata from C57BL6 and BALB/c mice infected with CVB3 Wt and pBRCVB3. (DOCX)
