# Correction: Mutations in the 5' NTR and the Non-Structural Protein 3A of the Coxsackievirus B3 Selectively Attenuate Myocarditogenicity

**DOI:** 10.1371/journal.pone.0137433

**Published:** 2015-08-31

**Authors:** Chandirasegaran Massilamany, Arunakumar Gangaplara, Rakesh H. Basavalingappa, Rajkumar A. Rajasekaran, Hiep Vu, Jean-Jack Riethoven, David Steffen, Asit K. Pattnaik, Jay Reddy

The following information is missing from the Funding section: This work was supported by National Institutes of Health grant HL114669.

The complete, correct Funding statement is as follows: This work was supported by the Nebraska Research Initiative Grant, and the National Institutes of Health (HL114669). CM is a recipient of a postdoctoral research fellowship grant awarded by the Myocarditis Foundation, NJ.

## References

[1] Massilamany C, Gangaplara A, Basavalingappa RH, Rajasekaran RA, Vu H, Riethoven J-J, et al (2015) Mutations in the 5’ NTR and the Non-Structural Protein 3A of the Coxsackievirus B3 Selectively Attenuate Myocarditogenicity. PLoS ONE 10(6): e0131052 doi: 10.1371/journal.pone.0131052 26098885 10.1371/journal.pone.0131052 PMC4476614

