# Supplementary figures and images for: Mutations in the 5’ NTR and the Non-Structural Protein 3A of the Coxsackievirus B3 Selectively Attenuate Myocarditogenicity
Source: PLoS One. 2015 Jun 22;10(6):e0131052. doi: 10.1371/journal.pone.0131052 (PMC4476614; doi:10.1371/journal.pone.0131052)

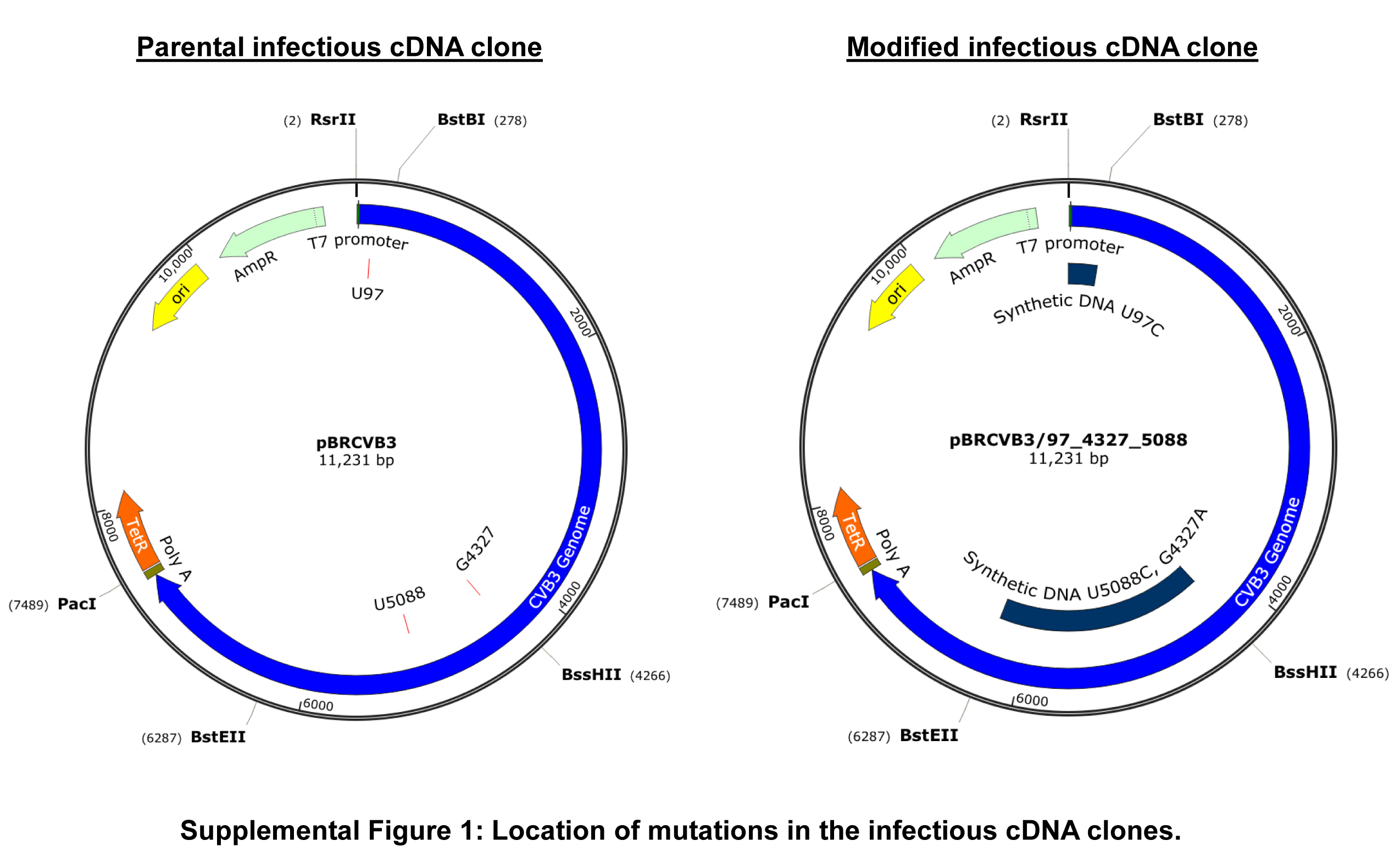

Supplement: S1 Fig — Left panel indicates the location of nts U, G and U at positions, 97, 4327 and 5088, respectively, in parental infectious cDNA clone. Right panel indicates the location of reverted nts U to C, G to A, and U to C, at positions, 97, 4327 and 5088, respectively, in the newly generated infectious cDNA clones. Vector maps were derived using SnapGene software. (TIF) [file pone.0131052.s001.tif]

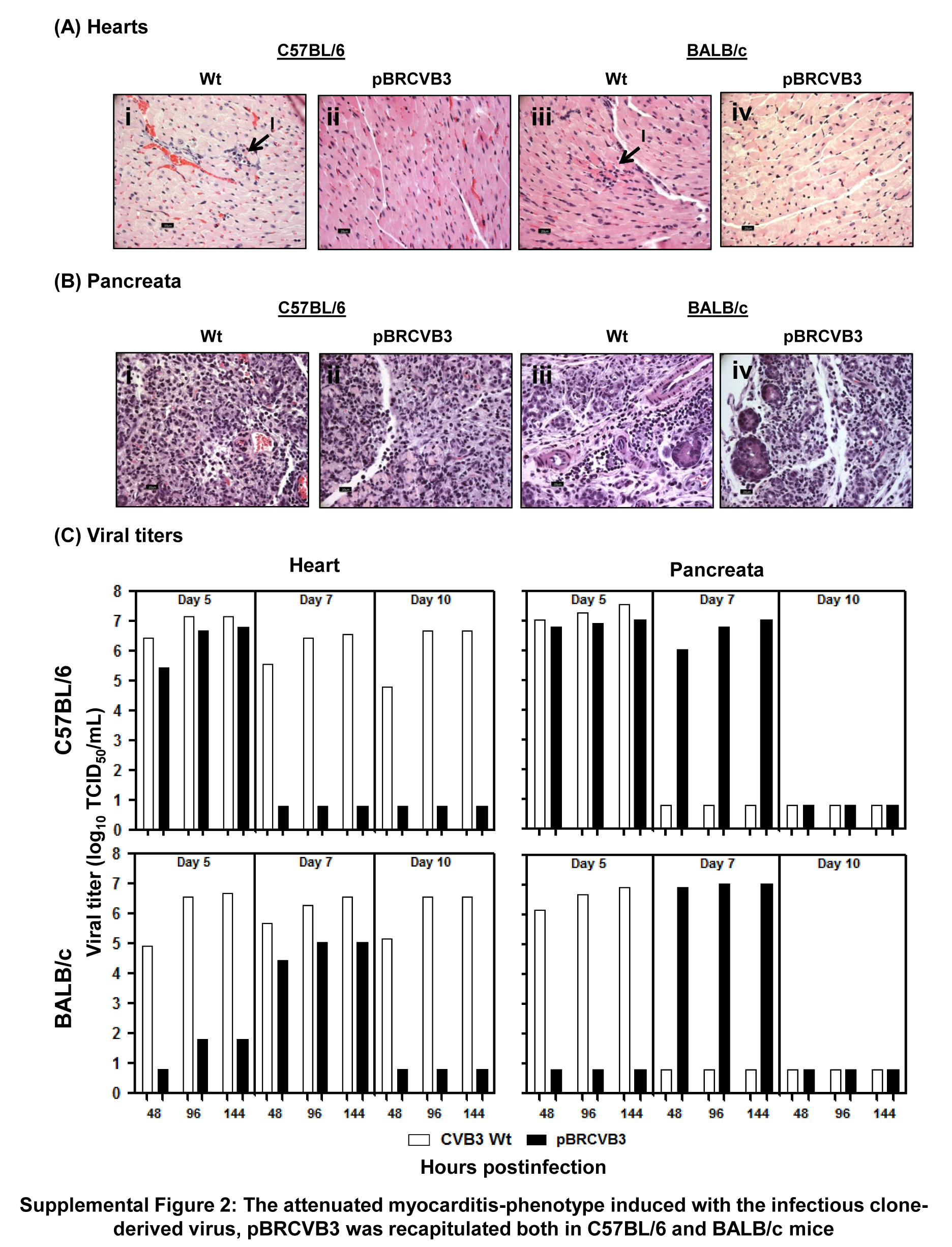

Supplement: S2 Fig — Groups of C57BL/6 and BALB/c mice were infected with Wt virus or pBRCVB3 virus. Animals were euthanized on days 5, 7, and 10 postinfection, and hearts and pancreata were collected for histological examination by H and E staining. (Figure A) Hearts. Section from C57BL/6 mice infected with Wt virus showing a necrotic fiber (arrow) surrounded by a few lymphocytes (i), as opposed to apparently normal heart from pBRCVB3 virus-infected animal (ii). Similarly, BALB/c mice showed hyper-eosinophilic necrotic fibers with pyknotic nuclei surrounded by a few lymphocytes (arrow; iii), whereas animals infected with pBRCVB3 virus had apparently normal hearts (iv). (Figure B) Pancreata. Four representative sections are shown, two each for C57BL/6 and BALB/c mice. C57BL/6: Wt virus-infected animal showing diffuse necrosis and inflammation (i); and pBRCVB3 virus-infected animal showing diffuse necrosis, inflammation, and mineralization (ii). BALB/c mice: Animal infected with Wt virus showing foci of lymphocytic inflammation in atrophied pancreas (iii), as opposed to diffuse inflammation with atrophied pancreas in pBRCVB3 virus-infected mouse (iv). Original magnification, 40x. n = 6 per group. (Figure C) Viral titers. Hearts and pancreata harvested from the above groups were processed for determining the viral titers as described in the methods section. Top panels: Heart and pancreata from C57BL/6 mice; bottom panels: Heart and pancreata from BALB/c mice. (TIF) [file pone.0131052.s002.tif]
