# Supplementary material for: Mutations in the 5’ NTR and the Non-Structural Protein 3A of the Coxsackievirus B3 Selectively Attenuate Myocarditogenicity
Source: PLoS One. 2015 Jun 22;10(6):e0131052. doi: 10.1371/journal.pone.0131052 (PMC4476614; doi:10.1371/journal.pone.0131052)
Supplement: S1 Table — (DOCX) [file pone.0131052.s003.docx]

**S1 Table:**

| Region | Nucleotide changes |
| --- | --- |
| 1. Comparison between pBRCVB3 and AC_M33854.1 [16] | |
| 5’NTR | 35G^+^; C97U; C610U |
| VP4 | G913A^*^ |
| VP2 | A1180G^*^; C1401G, Thr to Ser; G1402C, Thr to Ser; G1408A^*^ |
| VP3 | U2182C^*^; U2271A, Tyr to Phe |
| VP1 | A2690G, Glu to Lys |
| 2C | A4078G^*^; A4165G^*^; A4327G^*^ |
| 3A | C5088U, Pro to Leu |
| 1. Comparison between pBRCVB3 and AC_M16572.1 [18] | |
| 5’ NTR | 35G^+^; C97U; 274G^+^; C647U; U667C |
| VP4 | G788A, Gly to Arg |
| VP2 | A1180G^*^; A1272U, Asp to Val; U1273A, Asp to Val; C1401G, Thr to Ser; G1402C, Thr to Ser |
| VP3 | U1963C^*^; U2148C, Leu to Pro; G2201A, Val to Ile; G2289A, Cys to Tyr; G2438C, Glu to Gln |
| VP1 | A2520C, Asn to Thr; A2560G^*^; C2593U^*^; C2851U^*^; 3302A^-^, Phe to Ile; 3336A, Asp to Asn; A3346G^*^; C3358U, Ser to Leu; U3359C, Ser to Leu; C3360U, Ser to Leu |
| 2B | A3913G^*^; C4031G, Pro to Ala |
| 2C | A4177G^*^; A4327G^*^; U4582G, Gln to His; G4948U^*^ |
| 3A | U5051A, Phe to Ile; C5088U, Pro to Leu; A5249G, Met to Val |
| 3C^pro^ | U5560C^*^; G5588A, Glu to Lys; G5612A, Gly to Arg; G5621A, Gly to Arg; G5630U, Val to Leu; A5894U, Asn to Tyr |
| 3D^pol^ | U5943A, Val to Asp; A6007G^*^; U6015A, Val to Glu; G6142A^*^; A6212G, Arg to Val; G6213U, Arg to Val; U6247A^*^; C6341U, Arg to Cys; C6380U, His to Tyr; A6742G, Asn to Asp; U7026C, Val to Ala; U7073C^*^; C7086U, Ala to Val; C7087U, Ala to Val; A7265U, Ser to Thr; G7274C, Gly to Arg |
| 3’ NTR | 7314U^+^; C7319U; A7333; C7334U |
| 1. Comparison between pBRCVB3 and AC_JN048468.1 [21] | |
| 5’ NTR | C97U; G125A; G578A; C647U; U667C |
| VP4 | G788A, Gly to Arg |
| VP2 | A1180G^*^ |
| VP3 | G1861A^*^; U1963C^*^; G2201A, Val to Ile; C2430U, Thr to Ile; G2438C, Glu to Gln |
| VP1 | A2560G^*^; C2593U^*^; C2851U^*^; C3324U, Ala to Val; A3346G^*^ |
| 2C | A4177G^*^; A4327G^*^ |
| 3A | C5088U, Pro to Leu |
| 3D^pol^ | U7026C, Val to Ala; U7163C^*^ |
| 3’ NTR | C7334U |

^+^indicates additions; ^-^indicates deletions; and ^*^represents silent mutations.
