# Supplementary material for: Mutations in the 5’ NTR and the Non-Structural Protein 3A of the Coxsackievirus B3 Selectively Attenuate Myocarditogenicity
Source: PLoS One. 2015 Jun 22;10(6):e0131052. doi: 10.1371/journal.pone.0131052 (PMC4476614; doi:10.1371/journal.pone.0131052)
Supplement: S2 Table — (DOCX) [file pone.0131052.s004.docx]

**S2 Table:**

| Strain | Group | Incidence | Myocardial lesions |
| --- | --- | --- | --- |
| C57BL6 | CVB3 Wt | 5/6 (83.33) | 4.33 ± 1.02 |
|  | pBRCVB3 | 0/6 (0) | 0 |
| BALB/c | CVB3 Wt | 4/6 (66.67) | 1.83 ± 0.70 |
|  | pBRCVB3 | 0/6 (0) | 0 |

() represents percentages
