# Supplementary material for: Mutations in the 5’ NTR and the Non-Structural Protein 3A of the Coxsackievirus B3 Selectively Attenuate Myocarditogenicity
Source: PLoS One. 2015 Jun 22;10(6):e0131052. doi: 10.1371/journal.pone.0131052 (PMC4476614; doi:10.1371/journal.pone.0131052)
Supplement: S3 Table — (DOCX) [file pone.0131052.s005.docx]

**S3 Table:**

| Strain | Group | Incidence | Lesions | | | |
| --- | --- | --- | --- | --- | --- | --- |
|  |  |  | Atrophy | Inflammation | Necrosis | Mineralization |
| C57BL6 | CVB3 Wt^‡^ | 2/4 (50.00) | 2/4 (50.00) | 2/4 (50.00) | 1/4 (25.00) | 1/4 (25.00) |
|  | pBRCVB3 | 4/6 (66.66) | 4/6 (66.67) | 4/6 (66.67) | 0/6 (0) | 0/6 (0) |
| BALB/c | CVB3 Wt | 6/6 (100.0) | 5/6 (83.33) | 5/6 (83.33) | 2/6 (33.33) | 3/6 (50.00) |
|  | pBRCVB3 | 6/6 (100.0) | 6/6 (100.0) | 6/6 (100.0) | 0/6 (0) | 0/6 (0) |

() represents percentages**;** ^‡^ pancreas from two mice were not examined in this group, as the tissue was insufficient

for processing for H and E
